# Supplementary material for: WordCommentsAnalyzer: A windows software tool for qualitative research
Source: F1000Res. 2018 Sep 4;7:536. Originally published 2018 May 3. [Version 2] doi: 10.12688/f1000research.14819.2 (PMC6137411; doi:10.12688/f1000research.14819.2)
Supplement: Supplementary file 3 [file f1000research-7-17614-s0002.tgz › b2a79496-4ada-47c4-9682-a99ce867836d.zip!/Eight Iranian Health Journals/Yafteh _35.docx]

© Yafteh  . This work is licensed under CC BY 4.0 (https://creativecommons.org/licenses/by/4.0/).

Volume & Issue: دوره 12 - شماره 1

Title: بررسی عوامل مؤثر بر نتیجه امتحان جامع علوم پایه دانشجویان پزشکی دانشگاه علوم پزشکی لرستان

http://yafte.lums.ac.ir/article-1-310-fa.html

یکی از ارکان ضروری و اجتناب‌ناپذیر بهبود کیفیت نظام آموزشی به ویژه در دانشگاه‌ها، ارزیابی مداوم و مستمر وضعیت روند تحصیلی دانشجویان در مقاطع مختلف آموزشی می‌باشد. دوره‌ آموزشی علوم پایه پزشکی پیش‌زمینه پیشرفت تحصیلی و فهم دقیق مطالب دوره‌های بعدی پزشکی عمومی می‌باشد. دانشجوی پزشکی با استفاده از اطلاعات کسب شده در این دوره به حل مشکلات بالینی و تصمیم بالینی می‌پردازد. بنابراین به منظور شناسائی عوامل مؤثر در موفقیت دانشجویان در امتحان جامع علوم پایه دانشگاه علوم پزشکی لرستان مطالعه کنونی انجام گردید. مواد و روش ها: این پژوهش یک مطالعه توصیفی – تحلیلی است . جامعه آماری شامل کلیه دانشجویان پزشکی ورودی سال‌های 1380 تا 1383 شرکت کننده در آزمون جامع علوم پایه پزشکی بود. روش نمونه‌گیری به صورت سرشماری و تعداد نمونه‌ها 102 نفر بوده است. ابزار گردآوری اطلاعات شامل دو پرسشنامه مجزا است. اعتبار علمی پرسشنامه با استفاده از نظرات اعضاء هیئت علمی و اعتماد علمی پرسشنامه از طریق آزمون مجدد تعیین گردید. اطلاعات جمع‌آوری شده با استفاده از نرم‌افزار SPSS مورد تجزیه و تحلیل آماری قرار گرفت. در این مطالعه علاوه بر روش‌های آمار توصیفی جهت تحلیل اطلاعات از آزمون ANOVA، آزمون t مستقل و رگرسیون خطی استفاده شده است. بحث و نتیجه گیری: به منظور پیشرفت آموزش پزشکی و ایجاد تحول لازم در آن و با توجه به نقش مهم و اساسی دروس علوم پایه در پرورش شایستگی‌های پزشکان آینده، ایجاد گام‌های اصلاحی در آموزش پزشکی ضروری به نظر می‌رسد یافته ها: نتایج نشان داد که عوامل مؤثر بر نتیجه آزمون جامع علوم پایه در دروس مختلف به ترتیب زیر بوده اند: حجم سرفصل واحد درسی، نمره پایان ترم هر درس، نحوه آمادگی جهت آزمون، میزان اختصاص وقت جهت آمادگی برای آزمون، اهمیت تدریس واحد درسی در مقطع علوم پایه، کیفیت تدریس مدرس، میزان علاقمندی به واحد درسی، میزان اهمیت واحد درسی در امتحان جامع و سر انجام نحوه استفاده مدرس از وسایل سمعی و بصری و آزمایشگاهی.

Title: بررسی میزان غلظت عناصر سنگین در منابع تامین کننده آب شرب شهرستان الشتر در سال 1388

http://yafte.lums.ac.ir/article-1-306-fa.html

همگام با رشد صنعتی و اقتصادی و تولید انواع مختلف ترکیبات و مواد شیمیایی و غیره که بشر برای رفاه و آسایش خود با استفاده از منابع طبیعی به دست آورده است. در این راستا به طور ناخواسته موادی چون انواع فلزات سنگین و سمی را به طبیعت وارد می کند که هم برای محیط اطراف و هم برای خود مشکلات وخطرات جدی به همراه دارد. این مطالعه با هدف تعیین غلظت فلزات سنگین ( آرسنیک ، سرب، کادمیوم، کروم، جیوه ، روی )در کلیه چاه های تامین کننده آب شرب شهرستان الشتر در چهار فصل سال 1388 انجام گرفته است. بحث و نتیجه گیری: نتایج به دست آمده حاکی از آن است که میانگین غلظت سنجش شده در کلیه چاه های آب شرب الشتر پایین تر از حد استاندارد است. هیچ اختلاف معنی داری بین چهار مرحله نمونه برداری شده مشاهده نشد. نتیجه اینکه آب این چاه ها آلوده به فلزات سنگین نمی باشد. یافته ها: غلظت فلزات جیوه و روی و کادمیوم در همه دوره های نمونه برداری برابر صفر و میانگین غلظت فلزات آرسنیک ، سرب وکروم در چاه های آب شرب به ترتیب : 0033/ 0 ، 0788/ 0 و 01/ 0 میلی گرم در لیتر بود. مواد و روش ها: این مطالعه از نوع بررسی مقطعی بوده و به منظور بررسی غلظت فلزات آرسنیک ، سرب، کادمیوم،کروم، جیوه و روی در منابع آب شرب شهرستان الشتر به صورت منظم 20 نمونه از 5 حلقه چاه تامین کننده آب شرب شهرستان برداشت گردید.پس از انتقال نمونه ها به آزمایشگاه مطابق با روش ذکر شده در کتاب استاندارد متد 10 برابر تغلیظ شده و سپس عناصر مورد نظر در نمونه ها توسط دستگاه جذب اتمی اندازه گیری شد و با استفاده از نرم افزار های EXCEL،SPSS مورد تجزیه و تحلیل قرار گرفت.

Title: رابطه حمایت اجتماعی و سلامت روان در دانشجویان شهرستان خرم آباد (در سال 1388)

http://yafte.lums.ac.ir/article-1-305-fa.html

دانشجویان در دوران تحصیل ممکن است با مسائل و مشکلاتی مواجه شوند که سلامت روان آنها را تحت تأثیر قرار دهند. حمایت اجتماعی از عواملی محسوب می شود که در حفظ سلامتی افراد تأثیرگذار می باشد. در این مطالعه هدف آن است که به بررسی رابطه بین سلامت روان و حمایت اجتماعی پرداخته شود. مواد و روش ها: جامعه آماری این پژوهش را دانشجویان دانشگاه لرستان که در سال 1388 مشغول به تحصیل بودند، تشکیل داده بود. حجم نمونه 150 نفر انتخاب شد و داده ها با پرسشنامه مشتمل بر سه بخش مشخصات جمعیت شناختی و سلامت روان و حمایت اجتماعی، جمع آوری گردید. یافته ها: این پژوهش نشان داد بین منابع ساختاری و کارکردهای حمایت اجتماعی با سلامت روان رابطه معنی داری وجود دارد. در بین انواع کارکردهای حمایت اجتماعی، حمایت عاطفی و در بین منابع ساختاری حمایت اجتماعی، عضویت گروهی و پیوند قوی با سلامت روان رابطه معنی دار داشتند. دانشجویان تأثیر گذار باشند. بحث و نتیجه گیری: با توجه به وجود ارتباط بین حمایت اجتماعی و سلامت روان، توصیه می شود، مسئولین برنامه هایی را اتخاذ نمایند که موجبات ارتقای دریافت حمایت اجتماعی برای دانشجویان شوند و به این ترتیب در افزایش سلامت روان

Title: بررسی رضایت مندی شغلی اساتید هیات علمی دانشگاه علوم پزشکی لرستان

http://yafte.lums.ac.ir/article-1-304-fa.html

مقدمه: شناخت عوامل موثر در ایجاد رضایت شغلی می تواند در افزایش رضایت شغلی اعضای هیات علمی و ایجاد انگیزه در جهت پیشبرد اهداف آموزشی و پژوهشی مفید باشد . با توجه به این مهم پژوهش فوق با هدف تعیین میزان رضایت شغلی اعضای هیات علمی دانشگاه علوم پزشکی لرستان در سال 1386 انجام گرفت. یافته ها: در برر سی رضایت کلی 1/41% نمونه ها رضایت کامل داشتند. آزمون آماری کای دو بین رضایت کلی با حیطه های حقوق و مزایا(001/0= p ) ،فرصتهای ارتقاء(001/0= p ) ،نحوه سرپرستی (0001/0 p= ) ، امنیت شغلی (0001/0 p= ) ، شرایط فیزیکی محیط کار(001/0 p= ) ، وماهیت کار (001/0 p= ) ارتباط معناداری نشان داد. مواد و روش ها: پژوهش فوق توصیفی مقطعی که برروی اعضاء هیات علمی شاغل در دانشگاه علوم پزشکی لرستان که حداقل دو ترم تحصیلی فعالیت آموزشی داشتند ، انجام شد .ابزار گردآوری داده ها پرسشنامه بر گرفته از پرسشنامه رضایت شغلی هیات علمی دانشگاه علوم پزشکی اصفهان شامل 45 سوال در حیطه های حقوق و مزایا ، فرصتهای ارتقاء ،نحوه سرپرستی و ارتباطات ،امنیت شغلی ،شرایط فیزیکی و محیط ،ماهیت کار بصورت خود ایفاء تکمیل ، و بصورت توزیع فراوانی ،میانگین و انحراف معیار با نرم افزار spss ver 11.5 مورد تجزیه و تحلیل قرار گرفت. بحث و نتیجه گیری: با توجه به نتایج پژوهش مسئولین معاونت آموزشی و پژوهشی بایستی منابع رضایت و عدم رضایت شغلی را بخوبی شناسایی وبا ایجاد فرصتهایی برای ارتقاء و توسعه فردی ،انتخاب مدیران براساس شایستگی و با مشارکت اعضای هیات علمی، دقت در امر ارزشیابی صحیح عملکرد ، بکارگیری صحیح سیستم تشویق و تنبیه با استفاده از شیوه های نوین مدیریتی، ایجاد تسهیلات و امکانات رفاهی ، تعدیل در سقف موظفی افراد،دخالت اعضاء در تصمیم گیری ها رضایت شغلی را افزایش دهند.

Title: بررسی فعالیت بتاگالاکتوزیداز لاکتوباسیل های جدا شده از شیر و پنیر به روش های بیوشیمیایی و PCR

http://yafte.lums.ac.ir/article-1-303-fa.html

در برخی افراد مقدار ناکافی آنزیم بتاگالاکتوزیداز در لایه پرزدار مخاط رودهٔ کوچک ، سبب ایجاد اختلال عدم تحمل لاکتوز می گردد. این بررسی به منظور مشاهدهٔ وجود آنزیم بتاگالاکتوزیداز تولید شده توسط لاکتوباسیل های جدا شده از نمونه های لبنی (شیر و پنیر) بوده است. مواد و روش ها: نمونه های شیر و پنیر پاستوریزه و محلی از استان های لرستان، کرمانشاه، همدان، ایلام خریداری شد. لاکتوباسیل ها با کشت نمونه ها در محیط MRS و ویژگی های فنوتیپی شناسایی گردیدند. توانایی تولید بتاگالاکتوزیداز با روشهای X-galو ONPG و وجود ژن بتاگالاکتوزیداز با روش PCR بررسی گردید. یافته ها: از نمونه های بررسی شده131 لاکتوباسیل (13 جنس) جدا شد. که در محیط های حاوی X-gal کلنی های سبز، (در زمان های متفاوت) معرف وجود بتاگالاکتوزیداز، بدست آمد. با آزمایش ONPG، تمام لاکتوباسیل ها فعالیت بتاگالاکتوزیدازی را نشان دادند که در 25 نمونه مقدار آنزیم بیشتر از بقیه به دست آمد. بیشترین مقدار را لاکتوباسیلوس دلبروکی پنیر محلی کرمانشاه با 2010 واحد میلر در میلی لیتر داشت. در سوش استاندارد لاکتوباسیلوس پلانتاروم (RITCC 1273) و 29 نمونه لاکتوباسیلوس پلانتاروم ژن بتاگالاکتوزیداز بررسی و باند bp 399 مشاهده گردید. بحث و نتیجه گیری: با استفاده از لاکتوباسیل ها در فراورده های لبنی می توان به افراد فاقد تحمل لاکتوز کمک نمود. در ضمن لاکتوباسیل های با تولید بیشتر بتاگالاکتوزیداز، برای استفاده مناسب تر می باشند.

Title: ارزیابی حساسیت روش مستقیم درتشخیص تریکوموناس واژینالیس درمقایسه با محیط کشت دورسه

http://yafte.lums.ac.ir/article-1-300-fa.html

تریکوموناس واژینالیس تک یاخته تاژکداری است که در ناحیه ادراری - تناسلی زندگی کرده و باعث بیماری تریکومونیازیس در زنان می شود حدود 200 میلیون نفر درسراسر دنیا به آن مبتلا هستند. روش های متعددی با حساسیت و ویژگی های متفاوت در تشخیص این انگل به کار می روند که یکی از آن ها روش مستقیم با استفاده از ترشحات واژن است که نسبت به سایر روش ها روشی ساده، سریع و کم هزینه است. مواد و روش ها: متغیرهای مونوگرافیک به وسیله پرسشنامه که حاوی متغیرهای جمع آوری شده از 160 نفر از زنان مراجعه کننده به مرکز بهداشتی شهرستان خرم آباد بوسیله اسبیکولوم و دو سواپ پنبه ای نگهداری شده در محلول گلوگز از ترشحات واژن نمونه برداری شد که یکی ازنمونه ها در سرم فیزیولوژی و به صورت مستقیم در زیر میکروسکوپ برررسی شد و دیگری در محیط کشت دورسه کشت داده شد. یافته ها: از 160 زن مشکوک به تریکو مونیازیس به ترتیب 8/11% و 75/18% با روش مستقیم وکشت مثبت بودند که حساسیت روش مستقیم در این مطالعه5/63 درصد بود. یافته های ما بیانگر آن است که 30% زنان آلوده در گروه سنی 35-31 سال هستند. این گروه سنی دارای بیشترین فراوانی نسبی است . اکثر مبتلایان (43/0) بی سواد یا دارای تحصیلات ابتدائی بودند بحث و نتیجه گیری: حساسیت روش مستقیم نسبت به روش کشت (به عنوان استاندارد) 5/63 درصد بوده که حساسیت نسبتاً پایینی است. گرچه با کوتاه کردن فاصله زمانی نمونه برداری تا انجام ازمایش، استفاده از میکروسکوپیست های ماهر ونمونه های مختلف باعث اصلاح این روش می گردند ولی ما توصیه می کنیم که از متدهای با حساسیت بیشتر مانند کشت وPCR باید برای تشخیص استفاده شود

Title: ارزیابی و مقایسه بخش مدارک پزشکی بیمارستان های آموزشی دانشگاه های علوم پزشکی ایران و کرمانشاه بر اساس معیارهای استاندارد بین المللی، ایزو 2000 - 9001 سال1387

http://yafte.lums.ac.ir/article-1-299-fa.html

تعامل انسان ها با هم و زندگی در عصر حاضر به ویژه با رشد فن آوری، به اندازه ای پیچیده شده است که اگر برای همه موارد استاندارد معینی تعریف نشود و افراد ملزم به اجرای آن نگردند، زندگی طاقت فرسا می شود. از این رو، هرچه استاندارد سازی توسعه یابد و تعهد به رعایت استاندارد بیشتر شود، از پیشرفت و تعالی جامعه خبر می دهد. پژوهش حاضر با هدف ارزیابی و مقایسه بخش مدارک پزشکی بیمارستان های آموزشی دانشگاه های علوم پزشکی ایران و کرمانشاه بر اساس معیارهای استاندارد بین المللی، ایزو 2000-9001انجام گرفت. مواد و روش ها: پژوهش از نوع توصیفی می باشد که به صورت مقطعی انجام شد. جامعه آماری پژوهش بخش مدارک پزشکی 16 بیمارستان آموزشی وابسته به دانشگاه های علوم پزشکی ایران و کرمانشاه بود. ابزار گرد آوری داده ها پنج چک لیست بود که بر اساس بندهای اصلی استاندارد ایزو2000-9001 تنظیم گردید. داده ها با استفاده از مصاحبه و مشاهده عینی گردآوری شدند و با استفاده از شاخص های آمار توصیفی مورد تجزیه و تحلیل قرار گرفتند. یافته ها: در بیمارستان های دانشگاه ایران، معیار مدیریت منابع با 77درصد بیشترین امتیاز و معیار اندازه گیری، تحلیل و بهبود با 47 درصد کمترین امتیاز را داشت. در بیمارستان های کرمانشاه مدیریت منابع با 61 درصد بیشترین امتیاز و سیستم مدیریت کیفیت با 33 درصد کمترین امتیاز را داشت. میانگین کل معیارهای استاندارد در بیمارستان های دانشگاه ایران 60 درصد و کرمانشاه 46 درصد بود. بحث و نتیجه گیری: میزان تطابق نهایی سیستم مدارک پزشکی با معیارهای استاندارد ایزو 2000-9001 در بیمارستان های دانشگاه ایران نسبت به کرمانشاه بالاتر بود و میزان تطابق سیستم مدارک پزشکی در بیمارستان های کرمانشاه در حد ضعیف بود. بنابراین هماهنگ کردن بخش مدارک پزشکی با الزامات تعیین شده توسط استاندارد می تواند به ارتقای آن کمک کند.

Title: مقایسه شیوع سندرم درون گرایی و برون گرایی در بیماران بستری به علت خودکشی در بیمارستان های آموزشی خرم آباد در شش ماهه اول سال 1388

http://yafte.lums.ac.ir/article-1-311-fa.html

خودکشی موجب یک درصد موارد مرگ بوده و جزء هشت عامل عمده مرگ و میر است. یکی از عوامل زمینه ساز خودکشی اختلالات روانپزشکی است. دو مشکل درون گرایی و برون گرایی به عنوان مشکلات روانی آشکاری در افراد اقدام کننده به خودکشی شناخته شده اند. در این مطالعه میزان شیوع درون گرایی و برون گرایی در افراد اقدام کننده به خودکشی و ارتباط این دو با تعداد دفعات خودکشی و احتمال اقدام به خودکشی در آینده بررسی شده است. مواد و روش ها: در این مطالعه 200 نفر بیمار بستری شده به علت خودکشی ، با استفاده از پرسشنامه مورد بررسی قرار گرفتند تا میزان شیوع درون گرایی و برون گرایی در آنها مشخص شود. هم چنین تعداد دفعات اقدام به خودکشی و میانگین نمره احتمال اقدام مجدد به خودکشی در هر بیمار محاسبه شد و میانگین آنها در بین گروههای درون گرا و برون گرا و گروه طبیعی مقایسه گردید. بحث و نتیجه گیری: تمایل به درون گرایی و برون گرایی به طور شایعی در افراد اقدام کننده به خودکشی دیده می شود. خطر اقدام به خودکشی در آینده، در افراد متمایل به برون گرایی بیشتر از افراد متمایل به درون گرایی می باشد یافته ها: به طور خلاصه از 200 بیمار بررسی شده، 120 مورد (60 درصد) متمایل به برون گرایی، 70 مورد (35درصد) متمایل به درون گرایی و 10 مورد (5 درصد) طبیعی بودند. تعداد دفعات اقدام به خودکشی در گروههای متمایل به برون گرایی و درون گرایی و افراد طبیعی تفاوت معناداری نداشت. هر چند میانگین آن در گروه متمایل به برون گرایی بیشتر از گروه متمایل به درون گرایی و در هر دو گروه بیشتر از افراد طبیعی بود. احتمال اقدام مجدد به خودکشی به طور معناداری در گروه متمایل به برون گرایی بیشتر از گروه متمایل به درون گرایی بوده و در هر دو گروه بیشتر از افراد طبیعی بود.
